# Malnutrition in COVID-19 survivors: prevalence and risk factors

**DOI:** 10.1007/s40520-023-02526-4

**Published:** 2023-09-04

**Authors:** Matteo Tosato, Riccardo Calvani, Francesca Ciciarello, Vincenzo Galluzzo, Anna Maria Martone, Maria Beatrice Zazzara, Cristina Pais, Giulia Savera, Maria Camprubi Robles, Maria Ramirez, Francesco Landi, Gemelli Against COVID-19 Post-Acute Care Team, Francesco Landi, Elisa Gremese, Roberto Bernabei, Massimo Fantoni, Antonio Gasbarrini, Matteo Tosato, Carlo Romano Settanni, Serena Porcari, Francesca Benvenuto, Giulia Bramato, Vincenzo Brandi, Angelo Carfì, Francesca Ciciarello, Maria Rita Lo Monaco, Anna Maria Martone, Emanuele Marzetti, Carmen Napolitano, Vincenzo Galluzzo, Francesco Pagano, Cristina Pais, Sara Rocchi, Elisabetta Rota, Andrea Salerno, Matteo Tosato, Marcello Tritto, Riccardo Calvani, Maria Beatrice Zazzara, Lucio Catalano, Anna Picca, Giulia Savera, Mariaelena D’Elia, Damiano Biscotti, Roberto Cauda, Rita Murri, Antonella Cingolani, Giulio Ventura, Eleonora Taddei, Davide Moschese, Arturo Ciccullo, Massimo Fantoni, Leonardo Stella, Giovanni Addolorato, Francesco Franceschi, Gertrude Mingrone, Maria Assunta Zocco, Maurizio Sanguinetti, Paola Cattani, Simona Marchetti, Brunella Posteraro, Michela Sali, Alessandra Bizzarro, Alessandra Lauria, Stanislao Rizzo, Maria Cristina Savastano, Gloria Gambini, Grazia Maria Cozzupoli, Carola Culiersi, Giulio Cesare Passali, Gaetano Paludetti, Jacopo Galli, Fabrizio Crudo, Giovanni Di Cintio, Ylenia Longobardi, Laura Tricarico, Mariaconsiglia Santantonio, Tiziana Di Cesare, Mariateresa Guarino, Marco Corbò, Stefano Settimi, Dario Mele, Francesca Brigato, Danilo Buonsenso, Piero Valentini, Dario Sinatti, Gabriella De Rose, Luca Richeldi, Francesco Lombardi, Angelo Calabrese, Francesco Varone, Paolo Maria Leone, Matteo Siciliano, Giuseppe Maria Corbo, Giuliano Montemurro, Mariarosaria Calvello, Enrica Intini, Jacopo Simonetti, Giuliana Pasciuto, Veronica Adiletta, Carmelo Sofia, Maria Angela Licata, Gabriele Sani, Delfina Janiri, Alessio Simonetti, Marco Modica, Montanari Silvia, Antonello Catinari, Beatrice Terenzi, Luigi Natale, Anna Rita Larici, Riccardo Marano, Tommaso Pirronti, Amato Infante, Annamaria Paglionico, Luca Petricca, Barbara Tolusso, Stefano Alivernini, Clara Di Mario, Angelo Santoliquido, Luca Santoro, Antonio Nesci, Angela Di Giorgio, Alessia D’Alessandro

**Affiliations:** 1 Fondazione Policlinico Universitario “Agostino Gemelli” IRCCS, Largo Francesco Vito 1, 00168 Rome, Italy; 2 Department of Geriatrics, Orthopedics and Rheumatology, Università Cattolica del Sacro Cuore, 00168 Rome, Italy; 3 Abbott Nutrition, Research and Development, Camino de Purchil 68, 18004 Granada, Spain

**Keywords:** Nutritional status, GLIM criteria, Long Covid, Anorexia of aging, Geriatrics

## Abstract

**Background:**

Nutritional status is a critical factor throughout COVID-19 disease course. Malnutrition is associated with poor outcomes in hospitalized COVID-19 patients.

**Aim:**

To assess the prevalence of malnutrition and identify its associated factors in COVID-19 survivors.

**Methods:**

Study cohort included 1230 COVID-19 survivors aged 18–86 attending a post-COVID-19 outpatient service. Data on clinical parameters, anthropometry, acute COVID-19 symptoms, lifestyle habits were collected through a comprehensive medical assessment. Malnutrition was assessed according to Global Leadership Initiative on Malnutrition (GLIM) criteria.

**Results:**

Prevalence of malnutrition was 22% at 4–5 months after acute disease. Participants who were not hospitalized during acute COVID-19 showed a higher frequency of malnutrition compared to those who needed hospitalization (26% versus 19%, p < 0.01). Malnutrition was found in 25% COVID-19 survivors over 65 years of age compared to 21% younger participants (p < 0.01). After multivariable adjustment, the likelihood of being malnourished increased progressively and independently with advancing age (Odds ratio [OR] 1.02; 95% CI 1.01–1.03) and in male participants (OR 5.56; 95% CI 3.53–8.74). Malnutrition was associated with loss of appetite (OR 2.50; 95% CI 1.73–3.62), and dysgeusia (OR 4.05; 95% CI 2.30–7.21) during acute COVID-19.

**Discussion:**

In the present investigation we showed that malnutrition was highly prevalent in a large cohort of COVID-19 survivors at 4–5 months from acute illness.

**Conclusions:**

Our findings highlight the need to implement comprehensive nutritional assessment and therapy as an integral part of care for COVID-19 patients.

## Introduction

Nutrition is a major health determinant which is often overlooked in infectious diseases. Malnutrition, in particular protein-energy undernutrition, is the primary cause of immunodeficiency worldwide [1]. Infection in turn may lead to weight loss and (macro/micro-) nutrient deficiencies that further perturb immune response in a self-enhancing vicious cycle [1, 2].

In COVID-19, nutritional status plays a key role across all disease stages, especially in people at high risk of developing adverse outcomes (i.e., older adults and persons with multimorbidity) [3]. Poor nutrition may increase susceptibility to SARS-Cov-2 infection [4]. In mild-to-moderate COVID-19, the most prevalent symptoms, such as fever, fatigue, loss of appetite and alterations in smell and taste, are associated with reduced food intake, weight loss and higher risk of malnutrition [5]. In hospitalized COVID-19 patients, low levels of blood nutritional biomarkers (e.g. albumin and lymphocyte counts) are linked to worse outcomes [6, 7]. Recent evidence showed that malnutrition extended hospitalization in COVID-19 patients [8, 9]. Prolonged hospital stays and intensive care unit admission are associated with substantial loss of muscle mass and strength, reduced physical function and higher risk of malnutrition [10]. Following Sars-Cov-2 infection, catabolic processes and anorexia may be aggravated by excessive inflammatory response. These phenomena may further exacerbate malnutrition and lead to impaired recovery, loss of independence, disability, and reduced quality of life after hospital discharge [11]

Prevalence of malnutrition in hospitalized COVID-19 ranges from 14 to 70% depending on the study population, intensity of care, and screening/assessment tool used [12]. In a Chinese cohort of hospitalized older adults, 27.5% were at risk of malnutrition and 52.7% malnourished according to the Mini Nutritional Assessment (MNA) [13]. In a cohort of older adults from Italy, 77% were at nutritional risk using modified Nutritional Risk Screening 2002 and approximately 50% malnourished (according to Global Leadership Initiative on Malnutrition [GLIM] criteria) [14]. In this regard, both prevalence and severity of malnutrition were greater in intensive care compared to intermediate care and rehabilitation units. Similarly, in a cohort of French COVID-19 patients hospitalized in non-intensive medical units, the overall prevalence of malnutrition was 42.1% and rose to 66.7% in patients transferred to intensive care unit [15]. Comparable results were observed in a prospective observational cohort study (NUTRI-COV) conducted in Toulouse, in which 37.5% of COVID-19 inpatients were malnourished [16]. A recent systematic review and metanalysis showed that the pooled prevalence of malnutrition among hospitalized patients with COVID-19 was 49,1% and the risk of mortality in malnourished patients was ten times higher compared to well-nourished peers [17].

A large share of COVID-19 survivors reports persistent symptoms several weeks or months after Sars-CoV-2 infection, the so-called post-COVID syndrome or Long Covid [18, 19]. In this context, malnutrition may represent both a cause and a consequence of Long Covid and a useful metric to monitor recovery from acute disease [20, 21].

The aim of the present study was to assess the prevalence of malnutrition at 4–5 months after Sars-CoV-2 infection in a large sample of COVID-19 survivors attending a dedicated outpatient service. The association of malnutrition with clinical and functional characteristics was also investigated, with a particular focus on older adults.

## Materials and methods

Data for the present investigation were from the Gemelli Against COVID-19 Post-Acute Care (GAC19-PAC) project. GAC19-PAC is an initiative developed by the Department of Geriatrics, Neuroscience and Orthopedics of the Catholic University of the Sacred Heart (Rome, Italy). GAC19-PAC was conducted at a dedicated outpatient service which was established in April 2020 at the Fondazione Policlinico Universitario Agostino Gemelli IRCCS (Rome, Italy) to investigate long-term consequences of COVID-19 and their impact on health and quality of life [19–21]. Details about the post-COVID-19 outpatient service and patient evaluation were described elsewhere [22]

### Study Sample and data collection

The study population included adult subjects admitted to the post-COVID-19 outpatient service between April 2020 and November 2021.

Patients were offered a comprehensive medical assessment. A multidisciplinary approach was conducted to assess long-term consequences of SARS-CoV-2 infection [23] All clinical parameters, including medical history and medication inventory, lifestyle habits (e.g., smoking status and physical activity), education level and anthropometric measures were collected in a structured electronic data collection system. Smoking habit was categorized as current or never/former smoker. Regular participation in physical activity was considered as the engagement in leisure-time physical activity and/or exercise training at least twice weekly during the past year [24].

Both acute COVID-19 and persistent symptoms were collected on admission using a standardized questionnaire, as previously described [18].

COVID-19 severity was categorized as follows: (a) no hospitalization; (b) hospitalization not requiring supplemental oxygen; (c) hospitalization with oxygen supplementation; (d) hospitalization with high-flow oxygen supplementation; (e) hospitalization with intensive care unit (ICU) admission with invasive ventilation [25]

Time from COVID-19 diagnosis to study inclusion was calculated based on self-report.

### Nutritional assessment

Body weight was measured through an analog medical scale. Body height was measured using a standard stadiometer. Body mass index (BMI) was calculated as weight (in kilograms) divided by the square of height (in meters). Low BMI was defined as having a BMI below 20 kg/m^2^ in subjects younger than 70 years of age and below 22 kg/m^2^ if 70 years and older.

Body composition was measured in a fasting state using direct segmental multifrequency bioelectrical impedance (BIA) equipment (InBody S10, Seoul, Korea).  Appendicular skeletal mass (SM) index was calculated using validated BIA prediction equation (SM/height^2^) and expressed in kg/m^2^ [26, 27]. According to the European Working Group on Sarcopenia in Older People (EWGSOP) [28, 29], reduced muscle mass was identified as an appendicular skeletal mass index below 8.87 kg/m^2^ in males and 6.42 kg/m^2^ in females [27].

Data on appetite and dietary intake of study participants during acute COVID-19 were collected through a face-to-face interview by a registered dietitian.

Finally, according to the Global Leadership Initiative on Malnutrition (GLIM) criteria [30], participants were diagnosed as malnourished if they met at least one phenotypic criterion (low BMI and/or reduced muscle mass) and at least one etiologic criterion (reduced food intake and/or inflammation) (Table 1). The presence of inflammation was defined as having C-Reactive Protein (CRP) levels above 5 mg/L.

**Table 1:** Phenotypic and etiologic criteria for diagnosis of malnutrition according to GLIM definition

Characteristics			
Phenotypic criteria	Low BMI (kg/m^2^)	OR	Appendicular Skeletal Muscle Index (kg/m^2^)
	< 20 if < 70 years old		< 8.87 IF male
	< 22 if ≥ 70 years old		< 6.42 IF female
and			
Etiologic criteria	Reduced food intake	OR	Inflammation (CRP > 5 mg/L)

### Statistical analyses

Continuous variables were expressed as mean ± standard deviation (SD), categorical variables as frequencies by absolute value and percentage (%) of the total. Descriptive statistics were used to report clinical characteristics of the study population according to malnutrition status. The differences in proportions and means of covariates between study participants with and without malnutrition were assessed using Fisher’s Exact Test and t test statistics, respectively.

Logistic regression models were built to assess the association between clinical and functional characteristics and malnutrition status. Candidate variables to be included in the regression models were selected based on biological and clinical plausibility. To identify factors independently associated with malnutrition, we first estimated crude odds ratio (OR) and its 95% confidence interval (CI). A multivariable regression model was computed including all the variables that were associated with the outcome at α level of 0.05, after adjustment for age and gender. Model 1 included all clinical variables and COVID-19 symptoms potentially related with malnutrition. Model 2 was built adding COVID-19 severity scores.

All analyses were performed using SPSS software (version 11.0, SPSS Inc., Chicago, IL).

## Results

Between April 2020 and November 2021, 2100 COVID-19 survivors were admitted to the post-COVID-19 outpatient service. The present investigation included 1230 participants (mean age 54.6 ± 14.1 years; age range 18–86; 48% women) for whom all variables of interest were available.

**Fig. 1:**
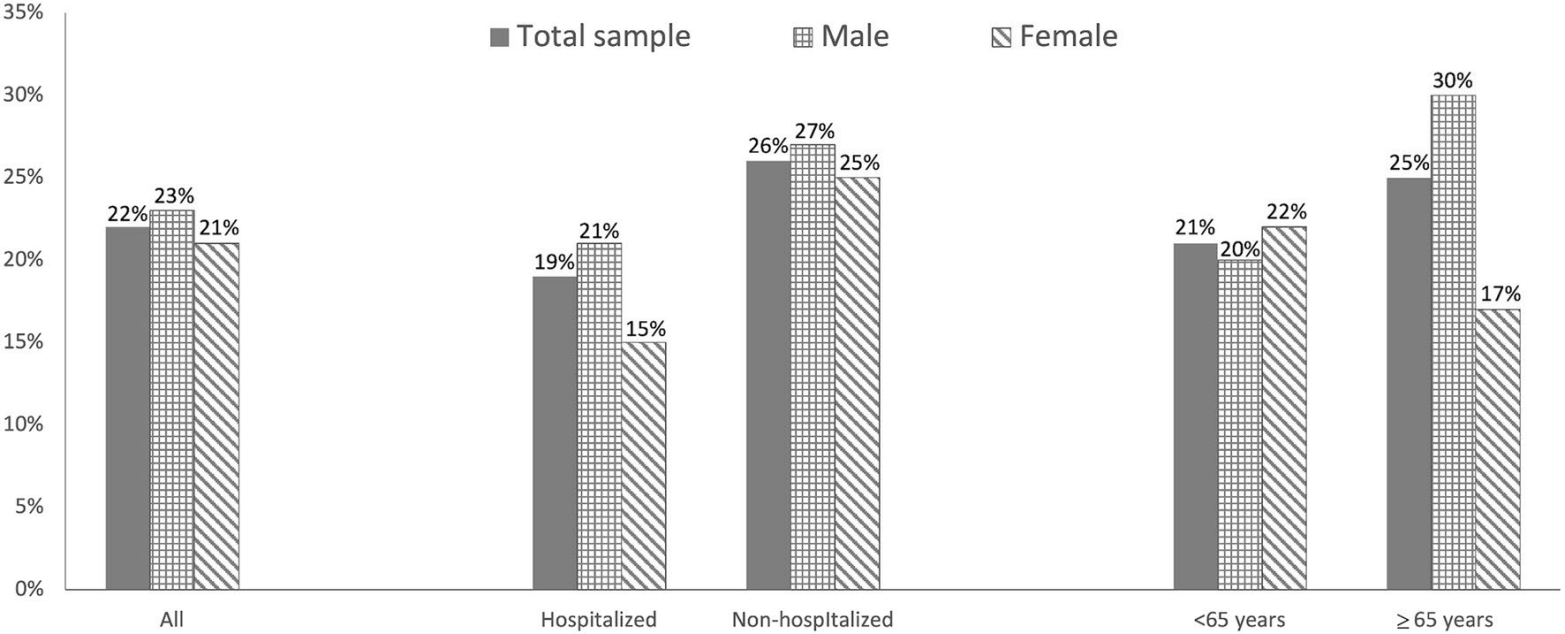
Prevalence of malnutrition identified by GLIM criteria according to gender, hospitalization and age group

The prevalence of malnutrition identified according to GLIM criteria was 22%, with no significant difference between males and females (23% vs 21%, p = 0.28). The average time from COVID-19 diagnosis to the inclusion in the study was significantly higher among participants with malnutrition versus non-malnourished peers (147±81 versus 131 ± 70, p = 0.01). Figure 1 shows the prevalence of malnutrition according to hospitalization and age groups. Interestingly, at the time of admission at post-COVID-19 outpatient service, participants who were not hospitalized during acute COVID-19 showed a higher rate of malnutrition compared to those who needed hospitalization (26% versus 19%, p < 0.01). Malnutrition was diagnosed in 25% of COVID-19 survivors older than 65 years, compared to 21% of younger participants (p < 0.01). In particular, in older participants, the prevalence of malnutrition was significantly higher in males compared to females (30% versus 17%, p < 0.001).

Characteristics of the study population according to malnutrition status are summarized in Table 2. Participants with malnutrition had lower BMI than those who were well-nourished (22.5 ± 3.5 versus 27.2 ± 4.7, p < 0.001). No differences in comorbidities and hematological parameters were observed. Notably, the prevalence of specific COVID-19-related symptoms during acute disease (loss of appetite, dysgeusia, myalgia, joint pain, smell disorders, headache and skin lesion) was significantly higher among malnourished participants compared to those who were not malnourished (Table 3).

**Table 2:** Characteristics of the study population according to malnutrition status identified by GLIM criteria

Characteristics	Whole Sample (n = 1230)	No malnutrition (n = 959)	Malnutrition (n = 271)	p
General and clinical characteristics				
Age (years)	54.6 ± 14.1	53.9 ± 16.1	54.8 ± 13.6	0.36
Gender				
Male	637 (52)	492 (51)	145 (53)	0.28
Female	593 (48)	467 (49)	126 (47)	
Education (years)	14.3 ± 4.5	14.6 ± 4.0	13.2 ± 4.5	0.55
Smoke habit	103 (9)	75 (9)	28 (11)	0.56
Physically active	630 (53)	489 (52)	141 (54)	0.35
Hypertension	389 (32)	314 (33)	75 (28)	0.07
Diabetes	117 (9)	98 (10)	19 (7)	0.07
Renal failure	22 (2)	20 (2)	2 (1)	0.10
COPD	71 (6)	50 (5)	21 (8)	0.07
Cancer	21 (2)	14 (2)	7 (3)	0.15
BMI (Kg/m^2^)	26.2±4.9	27.2±4.7	22.5±3.5	<0.001
Severity of COVID-19 during acute phase				
Not hospitalized	544 (45)	398 (41)	146 (54)	0.01
Hospital-no O_2_ support	120 (10)	97 (10)	23 (8)	
Hospital-O_2_ support	265 (22)	221 (23)	44 (16)	
Hospital-high-flow O_2_	225 (18)	188 (20)	37 (14)	
Hospital-Invasive Ventilation	76 (5)	55 (6)	21 (8)	
Length of hospital stay (days)	18.4 ± 11.9	18.2 ± 11.5	19.3 ± 13.7	0.39
Time from COVID-19 diagnosis (days)	134.8 ± 72.8	131.3 ± 69.9	147.2 ± 81.3	0.01
Hematological parameters				
Albumin	42.7 ± 3.0	42.6 ± 2.9	42.9 ± 3.4	0.21
Hemoglobin (g/dL)	14.1 ± 1.4	14.2 ± 1.4	14.0 ± 1.4	0.08
C-reactive protein (mg/L)	2.8 ± 6.1	2.7 ± 5.6	3.1 ± 7.4	0.26

Data are given as number (percent) for categorical variables; for continuous variables, means ± SD are reported

Physically active: engaged in physical activity routines at least twice a week

*BMI* body mass index

**Table 3:** COVID-19 symptoms reported during acute phase according to malnutrition status identified by GLIM criteria

Characteristics	Whole Sample (n = 1230)	No malnutrition (n = 959)	Malnutrition (n = 271)	p
Symptoms related to COVID-19 during acute phase				
Fatigue	1028 (84.1)	795 (83.3)	233 (86.6)	0.11
Cough	741 (60.4)	572 (59.8)	169 (62.4)	0.24
Short of breath	855 (69.9)	669 (70.2)	186 (68.9)	0.36
Loss of appetite	501 (41.3)	346 (36.7)	155 (57.8)	< 0.001
Dysgeusia	583 (47.6)	392 (41.0)	191 (70.7)	< 0.001
Myalgia	748 (61.2)	569 (59.7)	179 (66.5)	0.02
Joint pain	704 (57.6)	534 (56.0)	170 (63.2)	0.02
Smell disorders	573 (46.8)	400 (41.9)	173 (64.3)	< 0.001
Chest pain	463 (38.0)	366 (38.5)	97 (36.1)	0.25
Rhinitis	302 (24.8)	234 (24.6)	68 (25.4)	0.42
Diarrhea	387 (31.7)	297 (31.2)	90 (33.6)	0.25
Sore throat	355 (29.1)	280 (29.4)	75 (27.9)	0.33
Headache	622 (51.1)	475 (49.6)	150 (56.2)	0.03
Skin lesion	146 (12.0)	104 (11.0)	42 (15.8)	0.02
Number of symptoms	6.7 ± 3.3	6.5 ± 3.3	7.6 ± 3.0	< 0.001

Data are given as means ± SD for number of symptoms; number (percent) for all the other variables are reported

Logistic regression models were used to evaluate the association between clinical and functional characteristics during acute COVID and malnutrition. After multivariable adjustment (Model 2), the likelihood of being malnourished increased progressively and independently with advancing age (odds ratio [OR] 1.02; 95% CI 1.01–1.03) and the risk was significantly higher among male participants (OR 5.56; 95% CI 3.53–8.74). Malnutrition at the time of the study visit was associated with two symptoms reported by participants during the acute phase of COVID-19: loss of appetite (OR 2.50; 95% CI 1.73–3.62), and dysgeusia (OR 4.05; 95% CI 2.30–7.21) (Table 4).

**Table 4:** Unadjusted and adjusted association between clinical and functional parameters and malnutrition status identified by GLIM criteria

Characteristics	Unadjusted	Model 1	Model 2
	OR (95% CI)	OR (95% CI)	OR (95% CI)
Age (years)	1.04 (1.02–1.05)	1.02 (1.01–1.03)	1.02 (1.01–1.03)
Gender (Male)	1.09 (0.83–1.43)	5.62 (3.62–8.71)	5.56 (3.53–8.74)
Loss of appetite	2.37 (1.79–3.12)	2.50 (1.75–3.58)	2.50 (1.73–3.62)
Dysgeusia	3.47 (2.59–4.65)	3.81 (2.19–6.64)	4.05 (2.30–7.21)
Myalgia	1.34 (1.01–1.78)	1.04 (0.66–1.65)	0.98 (0.61–1.56)
Joint pain	1.34 (1.01–1.78)	1.17 (0.75–1.82)	1.26 (0.80–1.98)
Smell disorders	2.50 (1.88–3.31)	0.91 (0.52–1.59)	0.84 (0.48–1.48)
Headache	1.30 (0.99–1.71)	1.09 (0.73–1.61)	1.12 (0.75–1.67)
Skin lesion	1.52 (1.03–2.25)	1.29 (0.77–2.16)	1.20 (0.70–2.03)
Severity of COVID-19			
Home	1.0 (Reference)		1.0 (Reference)
Hospital-no O_2_ support	0.77 (0.48–1.26)		0.93 (0.51–1.70)
Hospital-O_2_ support	0.60 (0.42–1.03)		0.73 (0.43–1.23)
Hospital-high-flow O_2_	0.99 (0.56–1.78)		1.01 (0.57–1.80)
Hospital-Invasive Ventilation	1.05 (0.87–2.38)		1.95 (0.87–4.38)

*CI* confidence interval, *OR* Odds ratio

**Fig. 2:**
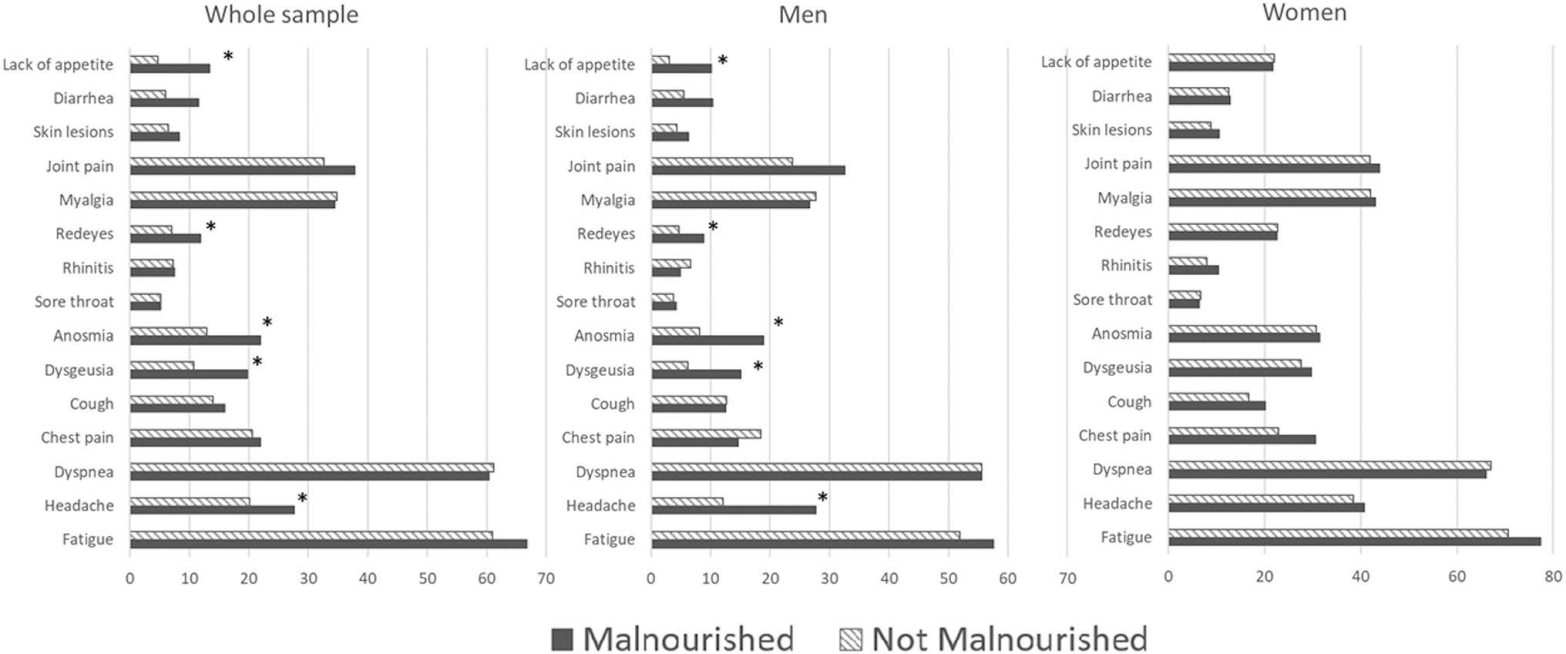
Prevalence of persisting symptoms according to sex and malnutrition status

In the post-acute phase, the most frequently reported symptoms were fatigue (65%), dyspnea (60%) and joint pain (42%). A gender-specific distribution of persistent symptoms was observed. Female participants showed a consistently higher prevalence of COVID19-related symptoms than males, regardless of malnutrition status. However, male participants with malnutrition reported a higher prevalence of anosmia, dysgeusia and lack of appetite than non-malnourished persons, while these differences were not present among female participants (Fig. 2).

## Discussion

In the present study, we explored the prevalence of malnutrition (according to GLIM criteria [31]) in a large sample of COVID-19 survivors at 4–5 months after acute disease. Several studies focused on the nutritional status of COVID 19 patients during hospital stay or after discharge [12]. However, to our knowledge, this is the first investigation to include a large sample of non-hospitalized COVID-19 survivors. Another important contribution from our study is the large number of participants included, although from a single center.

A wide range of malnutrition prevalence (14–70%) was reported in hospitalized COVID-19 patients during acute disease [12], with the majority being estimated at around 40% [17, 32].

Overall, the prevalence of malnutrition observed in our study sample was 22%, which indicates that malnutrition was present in a large share of COVID-19 survivors months after acute illness and may hinder full recovery in people with Long Covid. 

Our findings are in keeping with recent evidence from other post-COVID outpatient services which showed that malnutrition is one of the most common conditions among COVID-19 survivors [33]. At 23 days after hospital discharge, 54.7% of patients were at risk of malnutrition and 6.6% malnourished in a prospective observational study of 213 patients at a single center in Italy [5]. In a longitudinal study conducted on 91 hospitalized patients, 28.6% were malnourished at 30 days post-discharge, compared to 42.3% at admission [34]. In this cohort, the most relevant predictors of malnutrition were the need for high-flow oxygen therapy and/or invasive ventilation during hospitalization. In a cohort of 92 subjects attending a post-COVID recovery clinic at 3 months after Sars-CoV-2 infection, approximately half were at risk of malnutrition, which was associated with persistent gastrointestinal symptoms and reduced energy and protein intake [35]. In a French prospective cohort study on 288 hospitalized COVID-19 patients, 56.9% presented malnutrition at discharge according to GLIM criteria [36]. Of them, 47.2% showed persistent malnutrition at 30 days, and 36% at six months [20]. The most relevant factors for persistent malnutrition were intensive care unit admission and obesity.

In our study population, malnutrition risk increased with age, male gender, and was associated with loss of appetite and dysgeusia. Smell and taste dysfunction, and lack of appetite are highly prevalent symptoms related to COVID-19 that may have an impact on quality of life and overall health long after Sars-Cov-2 infection [18, 19, 37]. Poor taste is associated with lack of appetite and reduced dietary quality (low protein and high fat intake) in older adults [38].

Notably, poor appetite, and smell and taste disorders are traditionally associated with anorexia of aging, a major contributor to undernutrition and adverse health outcomes in older adults [39]. This corroborates the hypothesis that mechanisms involved in Long Covid may overlap with those of the aging process and aggravate pre-existing degenerative conditions [40, 41]. The strong association between poor appetite, dysgeusia and malnutrition risk found in our investigation underlines the need for a comprehensive nutritional assessment in COVID-19 patients throughout the disease course. In this regard, major clinical nutrition societies strongly recommend accurate and timely nutritional assessment and intervention to improve clinical outcomes in people at risk of malnutrition, especially in older adults, and in persons with multimorbidity [11, 42, 43]. Recently, European Society for Clinical Nutrition and Metabolism (ESPEN) developed a practical guidance for nutritional management of individuals with SARS-CoV-2 infection [44]. According to ESPEN recommendations, prevention, diagnosis and treatment of malnutrition should be considered as an integral part of COVID-19 patients’ continuum of care [44]. In this context, preliminary data from our post-COVID outpatient service showed that a comprehensive nutritional assessment together with the use of nutritional supplements (e.g., essential amino acids and derivatives) had a positive effects on nutritional status, functional recovery, and quality of life in COVID-19 survivors [45–47].

A sex-dependent distribution of acute and post-acute symptoms of COVID-19 was often observed. While a higher prevalence of acute symptoms (along with higher disease severity and mortality) was widely found in men, post-acute symptoms were more frequently reported by women [48, 49]. Our results are in line with recent systematic reviews and meta-analyses that have shown a sex-specific distribution of persisting symptoms, with more symptoms reported by women [48, 50]. However, in the present investigation, only male participants showed significant differences in the persistence of COVID19-related symptoms according to malnutrition status during the post-acute phase (Fig. 2). Specifically, anosmia, dysgeusia and inappetence were greater among malnourished participants, suggesting an enduring contribution of these symptoms to malnutrition status at all phases of the disease.

Limitations of the study include the lack of information on nutritional status before and at the time of acute COVID-19, and the lack of details on malnutrition severity. Moreover, this is a single center study with a large number of patients but without a control group (e.g., patients recovering from other infectious diseases). Patients with community-acquired pneumonia or patients with other viral diseases–such as herpes or chickenpox–can also have high rate of malnutrition, suggesting these findings could be not unique to COVID-19. However, the comprehensive medical assessment conducted during the study visit excluded the presence of any other acute infection.

## Conclusion

In the present study we showed that malnutrition was highly prevalent in a large cohort of COVID-19 survivors at 4–5 months from acute illness. Advanced age, poor appetite and taste disorders were the most relevant risk factors associated with malnutrition. Our findings highlight the need to implement comprehensive nutritional assessment and therapy as an integral part of COVID-19 patients’ care to optimize recovery from Sars-CoV-2 infection.

## Data Availability

The data that support the findings of this study are available on request from the corresponding author. The data are not publicly available due to their containing information that could compromise the privacy of the research participants.
